# Supplementary material for: Patterns of Intron Gain and Loss in Fungi
Source: PLoS Biol. 2004 Nov 30;2(12):e422. doi: 10.1371/journal.pbio.0020422 (PMC532390; doi:10.1371/journal.pbio.0020422)
Supplement: Table S1 — Also available at http://genes.mit.edu/NielsenEtAl/. (4.3 MB ZIP). [file pbio.0020422.st001.zip › NielsenEtAl/html/1239.html]

AN3220.1.NCU05585.1.MG05600.1.FG10483.1


```
 CLUSTAL W (1.82) Multiple Sequence Alignments - Introns Inserted


Sequence 1: AN3220.1	570 aa
Sequence 2: FG10483.1	559 aa
Sequence 3: MG05600.1	553 aa
Sequence 4: NCU05585.1	558 aa
Alignment Length: 574 aa
Number Identitical Residues: 279 aa
Alignment Score (without introns) 14191


MG05600.1 	MAG-PKKPVNVFKLKNLNVPPEVFNWRLWFAVLSFALLGAARGVDEGLISGAFNSKNFQD
NCU05585.1	MAGGVKKPVNIFKLKNLDEPKEVFNWRLWFAVLSFGLLGAARGVDEGLINGALKSKNFQD
FG10483.1 	MAGGVKKPVNIFKLKDLGEPAGVFNWRLWFAVVSFALLGAARGIDEGLISGSFNSKDFQE
AN3220.1  	MAGGPKQPLNVFRLNLGGAPKEVLNWRLWFAVLSFGLMGAARGVDEGLITGAFDSVDFQR
          	***. *:*:*:*:*:  . *  *:********:**.*:*****:*****.*::.* :** 

MG05600.1 	SINYKSYSTVDQANIKANVSAMVQIGSVGGSLF2AFLVCDRIGRIWAVRQLCAIWILGIG
NCU05585.1	SINYASYSTVEQANIKANISSMVQIGSVGGALI2-------IGRIWATRILCLLWMLGIA
FG10483.1 	KINYESYSEVEQANIKANVSAMVQIGSVGGALI2AFLVCDRIGRIWATRFLCTLWALGIV
AN3220.1  	TIDYESYSAVEQANIKANVTAMVQLGSVGGALF~AFLVCDRIGRIWAARVLCMLWVLGIA
          	.*:* *** *:*******:::***:*****:*: :   .. ******.* ** :* *** 

MG05600.1 	LFLGANGN--LGMIYAGRFIAGLGVG0Q~TPVVGPVYIAEI~APPEVRGLCTC~IFT~GF
NCU05585.1	IFMGSNGN--LSLVYFGRFVAGLGVG~Q0SPVVGPVYIAEI~APASIRGFCVC1TFT~GF
FG10483.1 	IFMIGGVNGNLGAIYAGRFICGLGVG~Q~TPVVGPVYIAEI~APAAIRGLCSC~MFT0GA
AN3220.1  	IFMGHGHS--LGAVYAGRLIAGFGVG~Q~TVVVGPVYLSEI0APAQVRGLCTC~IFT~GF
          	:*:  . .  *. :* **::.*:*** * : ******::** **. :**:* *  ** * 

MG05600.1 	VYLGIVLAYFANYGCQINMGDNTPARW~MVPTSLHIMFATIIFCLSFMQYESPRFFIKQG
NCU05585.1	VYLGIVLAYFTNYGCQLHLAE-TPARW~MVPTSLHIIFAGLILLLTFLQYESPRYLVKTG
FG10483.1 	VYIGIVLAYFTNYGAHLNMSDHTHNQW~LVPTSLHIMMSGIIFILMFFQSESPRYLVKQG
AN3220.1  	VYLGIVLAYFANYGCEVNMGDNTHKRW0EVPTSLHIMFAGLIFLLSFLQLESPRFLIKRS
          	**:*******:***..:::.: *  :*  *******::: :*: * *:* ****:::* .

MG05600.1 	KHEAAQYNMARLRNLPEDHPYVLNEIAAIEMAHQEEMEATKGTGFWGMLKEMFTVPSNLY
NCU05585.1	KSDKALHVMSRLRHLPPDHDYVRREINAITLSHHEEMEASKGAGWLGILKETFTVRSNLY
FG10483.1 	KPEEAAKVLGRLRQQSPESDYVVRQVAEIQAALDHELEATKGVGFLGKVKELLFVPSNLY
AN3220.1  	RYEEALVNLSKLRGLPTDHEYVLEELNGIRAAHEAEMEATMGLGWLGVLKEALLVPSNLY
          	: : *   :.:**  . :  ** .::  *  : . *:**: * *: * :** : * ****

MG05600.1 	RIWLALMAQIMSQ~WSGAGSITLYAPDLFKLLGIAGTNETLLVTAVFGIVKLIAAIICAL
NCU05585.1	RVGLTLGAQVMGQ~WSGAGSITIYAPDLFELLGLTGTKESLLVTAIFGIVKLVAAIVCAL
FG10483.1 	RLYMSSMVQFLSQ~WSGAGSITLYAPDLFKIMGIVGKQEGLLVTAVFGIVKLIAAVICAL
AN3220.1  	RLYLAAMVQILSQ0WSGAGSITVYAPDLFSLLGIRGSEEGLLVTAVFGLVKLAAAVICAL
          	*: ::  .*.:.* ********:******.::*: *.:* *****:**:*** **::***

MG05600.1 	FLVDVIGRKRSLLIGISLQAVSMIYVASFLTAVPKLGVDEALAMND--AQLGASKGAIAM
NCU05585.1	FLVDFIGRKRSLLLGIACQAISMIYIAAFLSATPELGNEEGYVIPANSGKLRASRGAIVM
FG10483.1 	FLVDVIGRKRALLIGITLQTIAMIYVASFLTKIPQLGVDEDFELPA--ADKGASRGAIAM
AN3220.1  	FLVDVIGRKRALLIGITLQAISMIYIAAFLTDTPEMGVDDSYILPE--SKKGSSRGAIAM
          	****.*****:**:**: *:::***:*:**:  *::* ::   :    ..  :*:***.*

MG05600.1 	IYISGFGWALGWNSMQYLLTSELFPLRIRALATSMAMTFHFANQYGNARALPNMLLPFGK
NCU05585.1	IYLSGIGWALGWNSMQYLLSSEIFCLRLRAFCSSLAMCLHFANQYGSARAVPDMLLPVRE
FG10483.1 	IYVSGFGWALGWNSMQYLLTAELFPLRIRALATSWAMTLHFANQYGNSRAVPNMLLPVAK
AN3220.1  	IYISGVGWALGWNSMQYLLTAELFPLRIRAMATSTAMTLHFANQYGNARAVPNMLLPTSH
          	**:**.*************::*:* **:**:.:* ** :*******.:**:*:****  .

MG05600.1 	GGINPNGSFWTFAIITILGGFWVWFFVPETAGRSLESMDRLFALPWYRIGRYGNADAEEQ
NCU05585.1	GGISGQGTFWFFSAVTILGGVWVWFFVPETASRSLESMDRLFSLPWWQIGRYGNREAEAL
FG10483.1 	GGISPKGTFWCFAAVTFVGGLWVYFSVPETSGRSLENMDELFELPWYKIGLHGNKHVEEL
AN3220.1  	GGIDPKGTFWCFAAVTILGGLWVWLSIPETKGRTLESMDALFQLPWWKIGLYGNRDAELR
          	***. :*:** *: :*::**.**:: :*** .*:**.** ** ***::** :** ..*  

MG05600.1 	DAVYKTKEEEAGVSS------GAHVEKTREIA----------
NCU05585.1	DEAERVGGEKEGEMMS---RSGSHVEEEKGMAYAAERKV---
FG10483.1 	DEARDEKKRYADEAN----ATGIELEHQRKQDA---------
AN3220.1  	DMENEKVGGHVDTRNEMEMATVTVGDEEKKAEGRAENVERVQ
          	*          .   . .  :    :. :     :.     .
```
